# Multidimensional psychosocial correlates of food addiction status and symptom count in Chinese candidates for bariatric surgery: a cross-sectional study

**DOI:** 10.3389/fpsyg.2026.1942332

**Published:** 2026-09-30

**Authors:** Yuyang Zhang, Ting Xu, Yacong Zhao, Luyao Liu, Ziyan Lin, Linwei Li, Jianzhong Di

**Affiliations:** Department of Bariatric and Metabolic Surgery, Shanghai Jiao Tong University School of Medicine Affiliated Sixth People’s Hospital, Shanghai, China

**Keywords:** bariatric surgery, body appreciation, depression, family appearance pressure, food addiction, obesity

## Abstract

**Objective:**

To examine the associations of sociocultural appearance pressure, depressive and anxiety symptoms and body appreciation with food addiction status and symptom count among Chinese candidates for bariatric surgery.

**Methods:**

This cross-sectional study included 240 candidates for bariatric surgery. Food addiction status and symptom count were assessed using the Yale Food Addiction Scale 2.0 (YFAS 2.0); psychosocial measures included the depressive symptoms (PHQ-9), anxiety (GAD-7), body appreciation (BAS-2), sociocultural attitudes toward appearance (SATAQ-4). Logistic regression was used to analyze food addiction status, and negative-binomial regression was used to analyze food addiction symptom count. The primary analysis used the full Model 3, which simultaneously included demographic, lifestyle and psychological variables. Hierarchical comparisons of Models 1 and 2 were treated as exploratory analyses.

**Results:**

Among the 240 participants, 95 (39.6%) met the criteria for food addiction. In the full logistic model, BMI (OR per SD = 1.549, 95% CI 1.094–2.193; *p* = 0.014) and family pressure (OR per SD = 1.516, 95% CI 1.030–2.232; *p* = 0.035) were positively associated with food addiction status. PHQ-9 (OR per SD = 1.421, 95% CI 0.886–2.278; *p* = 0.145) and BAS-2 (OR per SD = 0.719, 95% CI 0.511–1.011; *p* = 0.058) were not statistically significant. In the full negative-binomial model, family pressure (IRR per SD = 1.171, 95% CI 1.023–1.340; *p* = 0.022) and PHQ-9 (IRR per SD = 1.284, 95% CI 1.087–1.518; *p* = 0.003) were positively associated with symptom count, whereas BAS-2 was inversely associated (IRR per SD = 0.883, 95% CI 0.785–0.992; *p* = 0.037). Formal tests did not distinguish the coefficients for the three pressure sources. The apparent AUC of the logistic model was 0.761, and the optimism-corrected AUC was 0.707.

**Conclusion:**

Associations differed by outcome. BMI and family pressure were associated with food addiction status, whereas family pressure, depressive symptoms and body appreciation were associated with symptom count. Family pressure was the only pressure source with a statistically significant independent coefficient in the full models, but its coefficient did not differ significantly from peer or media pressure. These cross-sectional findings describe associations and require prospective research and external validation.

## Introduction

Food addiction (FA) is a proposed phenotype of addictive-like eating characterized by compulsive intake of highly rewarding foods, impaired control over eating and persistent eating behaviors (Carter et al., 2016; Sanchez et al., 2022; Butt et al., 2023). A systematic review and meta-analysis indicated that FA is common among candidates for bariatric surgery, with a pooled preoperative prevalence of 32% (Praxedes et al., 2023). FA may be associated with more severe eating disturbances, greater psychological distress, and poorer postoperative behavioral management, leading to insufficient weight loss or weight regain (Farias Tavares da Silva et al., 2026; Brunault et al., 2016; Benzerouk et al., 2018; Sevinçer et al., 2016; Ben-Porat et al., 2021). Therefore, identifying preoperative psychosocial factors associated with FA may be important for improving preoperative assessment and individualized intervention among candidates for bariatric surgery.

Food addiction is generally considered to have a complex psychosocial basis. Negative emotional states such as depression and anxiety are common in individuals with FA and may be linked to FA symptom burden through pathways including emotional eating, reward seeking and self-regulation difficulties (Açik and Ülger, 2025). Meanwhile, body image is also an important dimension in understanding FA. Negative body image may exacerbate eating disorders and weight-related distress, whereas positive body image, particularly body appreciation, may represent a protective psychological resource associated with lower eating pathology and better psychological adaptation (Perry et al., 2025; Linardon et al., 2022). In addition to individual psychological factors, sociocultural appearance pressure may also play an important role in FA. Appearance pressure from family, peers and the media, as well as the internalization of thin or muscular ideals, are recognized as key sociocultural sources of body dissatisfaction and eating disorders (Tanguay et al., 2025). However, the relationship between negative emotional states, body image perspective and different sources of appearance pressure and FA remains insufficiently studied in the high-risk population of patients with obesity seeking bariatric surgery.

Therefore, we examined the associations of FA status and symptom count with demographic and lifestyle factors, body mass index (BMI), appearance pressure, appearance-ideal internalization, depressive and anxiety symptoms, and body appreciation among Chinese candidates for bariatric surgery. Model 3 was used as the primary fully adjusted model and simultaneously included all candidate predictors. Hierarchical comparisons of Models 1 and 2 were retained as exploratory analyses of changes in model fit across conceptual variable blocks rather than as the basis for primary inference.

## Methods

### Participants

This cross-sectional study included patients who were scheduled to undergo bariatric surgery at Shanghai Sixth People’s Hospital between June 8, 2023, and December 29, 2025, and who completed a preoperative psychological assessment. Of 287 questionnaire records, 240 were retained for analysis; 26 were excluded because they failed the attention check, and 21 questionnaires were completed in less than 15 min. A completion time of <15 min was defined as an excessively short response time. The 240 questionnaires contained no missing data, and no statistical imputation was performed. Each participant was assigned a unique identifier for data linkage, and their information was kept confidential. The study was approved by the hospital Institutional Ethics Committee (Approval No: 2020–219-(1)).

### Questionnaires and scales

All questionnaires were administered via an online survey platform^1^. Demographic information, including sex, age, smoking history and alcohol use history, was collected using the Wenjuanxing platform. Height and weight were obtained from the preoperative clinical records and were measured by two clinicians as part of routine preoperative care. Body mass index (BMI) was calculated as weight in kilograms divided by height in meters squared (kg/m^2^).

The Yale Food Addiction Scale 2.0 (YFAS 2.0) was used to assess symptoms of food addiction (Gearhardt et al., 2016). The scale consists of 35 items rated on an 8-point Likert scale (0 = never, 7 = every day). Each item was dichotomized according to pre-established thresholds (0 = threshold not met, 1 = threshold met). Item scores were then aggregated into 11 symptom criteria; each criterion was scored as 1 if at least one corresponding item met the threshold, and 0 otherwise. The symptom count total score ranges from 0 to 11. The 12th criterion—clinical significance (functional impairment or distress)—was not counted toward the symptom total but was used for diagnostic classification. Diagnostic categories were defined as follows: no food addiction (fewer than 2 symptoms or absence of clinical significance); mild food addiction (2–3 symptoms with clinical significance); moderate food addiction (4–5 symptoms with clinical significance); and severe food addiction (≥6 symptoms with clinical significance). The complete Chinese version of YFAS 2.0 has undergone psychometric evaluation in Chinese-speaking samples, and the internal consistency of the 11 binary symptom criteria in this sample was KR-20 = 0.873.

The Patient Health Questionnaire-9 (PHQ-9) was used to assess depressive symptoms (Kroenke et al., 2001). The scale consists of 9 items rated on a 4-point Likert scale (0 = not at all, 1 = several days, 2 = more than half the days, 3 = nearly every day), with total scores ranging from 0 to 27. Scores of 0–4 indicate no depressive symptoms, 5–9 indicate mild depression, 10–14 indicate moderate depression, 15–19 indicate moderately severe depression, and 20–27 indicate severe depression. A total score of ≥10 was considered indicative of clinically significant depressive symptoms. The validated Chinese version was administered (Wang et al., 2014).

The Generalized Anxiety Disorder-7 (GAD-7) was used to assess anxiety symptoms (Spitzer et al., 2006). The scale consists of 7 items rated on a 4-point Likert scale (0 = not at all, 1 = several days, 2 = more than half the days, 3 = nearly every day), with total scores ranging from 0 to 21. Scores of 0–4 indicate no anxiety, 5–9 indicate mild anxiety, 10–14 indicate moderate anxiety, and scores of 15 or higher indicate severe anxiety. A total score of ≥10 was considered positive for clinically significant anxiety symptoms. The validated Chinese version was administered (Tong et al., 2016). In the present study, PHQ-9 and GAD-7 scores were analyzed primarily as continuous variables. Sample-specific Cronbach alpha values were 0.861 for PHQ-9 and 0.927 for GAD-7.

The Body Appreciation Scale-2 (BAS-2) was used to assess individuals’ positive attitudes toward and acceptance of their bodies (Tylka and Wood-Barcalow, 2015). The scale consists of 10 items rated on a 5-point Likert scale (1 = never, 5 = always). The total score was calculated as the mean of all items, with higher scores indicating greater levels of body appreciation. The validated Standard Chinese version was administered (Swami et al., 2016). Sample-specific Cronbach alpha was 0.913.

The Sociocultural Attitudes Towards Appearance Questionnaire-4 (SATAQ-4) was used to measure individuals’ internalization of sociocultural standards of beauty and ideal body shape, as well as perceived external pressure (Schaefer et al., 2015). The scale consists of 22 items rated on a 5-point Likert scale (1 = completely disagree, 5 = completely agree). It comprises five subscales: thin-ideal internalization, muscular internalization, family pressure, peer pressure, and media pressure. The validated Standard Chinese version was administered (Ma et al., 2023). Sample-specific Cronbach alpha values for the five subscales were 0.816, 0.866, 0.798, 0.843, and 0.894, respectively, all within an acceptable range.

### Statistical analysis

Analyses were performed using R version 4.5.1. Continuous variables are summarized as mean ± SD and categorical variables as n (%). Continuous variables were compared using independent-samples t tests or Wilcoxon rank-sum tests, and categorical variables using chi-square tests or Fisher’s exact tests.

The primary outcome was binary FA status (absent/present), and the secondary outcome was YFAS 2.0 symptom count (0–11). Logistic regression was used for FA status. A Poisson model was fitted for symptom count; its Pearson dispersion statistic was 2.495, supporting the use of negative-binomial regression. Odds ratios (ORs), incidence rate ratios (IRRs) and 95% confidence intervals (CIs) are reported.

The primary analysis for both outcomes used the full Model 3, which simultaneously included age, sex, BMI, drinking, smoking, family pressure, peer pressure, media pressure, thin-ideal internalization, muscular internalization, GAD-7, PHQ-9 and BAS-2. Model 1 included only age, sex, BMI, drinking and smoking; Model 2 further added family pressure, PHQ-9, and BAS-2. Models 1 and 2 were retained only as exploratory hierarchical comparisons to describe changes in model fit across conceptual variable blocks, rather than to select predictors according to univariable analyses or statistical significance. Nested models were compared using Akaike Information Criterion (AIC), Bayesian Information Criterion (BIC) and likelihood-ratio tests. The area under the receiver operating characteristic curve (AUC) and the Brier score summarized the performance of the primary logistic model, which underwent internal validation with 1,000 bootstrap resamples.

To compare different sources of appearance pressure, an omnibus Wald test was used in primary Model 3 to assess whether the family pressure, peer pressure, and media pressure coefficients were equal, followed by pairwise comparisons with 95% CIs and Benjamini–Hochberg correction. Spearman correlations and variance inflation factors (VIFs) were used to assess overlap among predictors. Model adequacy was evaluated using overdispersion, influence, and residual diagnostics.

All tests were two-sided, with *p* < 0.05 considered statistically significant. Given the cross-sectional design, regression results were interpreted only as statistical associations rather than causal effects.

## Results

### Basic characteristics of the study subjects

A total of 240 patients seeking bariatric surgery were included in this study, of whom 95 (39.6%) were assessed as having food addiction (FA) and 145 as non-food addiction (NFA). Differences were observed between the two groups in BMI, psychological distress, body appreciation, and sociocultural appearance pressure (Table 1). Compared with the NFA group, the FA group had a higher BMI (40.18 ± 6.87 vs. 37.97 ± 6.36, *p* = 0.011), higher PHQ-9 scores (9.43 ± 5.82 vs. 6.70 ± 5.29, *p* < 0.001), and higher GAD-7 scores (6.92 ± 5.79 vs. 4.43 ± 4.60, *p* < 0.001). Meanwhile, the FA group scored lower on the BAS-2 than the NFA group (3.23 ± 0.87 vs. 3.52 ± 0.84, *p* = 0.011). Regarding sociocultural appearance pressure, the FA group reported higher family pressure, peers pressure, media pressure, and total sociocultural appearance pressure scores than the NFA group (all *p* < 0.01). These findings suggest that patients with FA not only have a higher BMI but also experience greater psychological distress, higher appearance pressure, and lower levels of body appreciation.

**Table 1:** Baseline characteristics of participants according to food addiction status.

**Variable**	**Overall (N = 240)**	**NFA (n = 145)**	**FA (n = 95)**	**P**	**SMD**
Age, years	30.40 ± 8.22	29.86 ± 8.49	31.23 ± 7.76	0.205	0.169
Sex, *n* (%)				0.256	0.168
Male	77 (32.1)	42 (29.0)	35 (36.8)		
Female	163 (67.9)	103 (71.0)	60 (63.2)		
Weight, kg	110.47 ± 23.93	107.03 ± 23.59	115.72 ± 23.61	0.006	0.368
Height, cm	168.16 ± 8.70	167.38 ± 9.08	169.36 ± 8.00	0.084	0.232
BMI, kg/m^2^	38.84 ± 6.64	37.97 ± 6.36	40.18 ± 6.87	0.011	0.334
PHQ-9	7.78 ± 5.65	6.70 ± 5.29	9.43 ± 5.82	<0.001	0.492
GAD-7	5.42 ± 5.23	4.43 ± 4.60	6.92 ± 5.79	<0.001	0.475
BAS-2	3.41 ± 0.86	3.52 ± 0.84	3.23 ± 0.87	0.011	0.338
Thin-ideal internalization	3.34 ± 0.94	3.23 ± 0.94	3.50 ± 0.92	0.027	0.294
Muscular internalization	2.62 ± 0.97	2.51 ± 0.91	2.79 ± 1.04	0.029	0.286
Family pressure	3.74 ± 0.88	3.58 ± 0.91	3.98 ± 0.77	<0.001	0.483
Peer pressure	3.52 ± 0.92	3.37 ± 0.93	3.74 ± 0.87	0.003	0.402
Media pressure	3.26 ± 1.07	3.07 ± 1.05	3.55 ± 1.05	0.001	0.457
Drinking, *n* (%)				0.044	0.286
No	213 (88.8)	134 (92.4)	79 (83.2)		
Yes	27 (11.2)	11 (7.6)	16 (16.8)		
Smoking, *n* (%)				0.089	0.245
No	207 (86.2)	130 (89.7)	77 (81.1)		
Yes	33 (13.8)	15 (10.3)	18 (18.9)		

Values are presented as mean ± SD or n (%). FA, food addiction; NFA, non-food addiction; BMI, body mass index; PHQ-9, Patient Health Questionnaire-9; GAD-7, Generalized Anxiety Disorder-7; BAS-2, Body Appreciation Scale-2; SMD, standardized mean difference.

### Food addiction status: full logistic model

In the full logistic model, BMI and family pressure were positively associated with FA status (Table 2). Per SD, the OR was 1.549 (95% CI 1.094–2.193; *p* = 0.014) for BMI and 1.516 (95% CI 1.030–2.232; *p* = 0.035) for family pressure. PHQ-9 and BAS-2 were not statistically significant.

**Table 2:** Full logistic model for food addiction status.

**Predictor**	**OR/unit (95% CI)**	**OR/SD (95% CI)**	**P**
Age	1.033 (0.995–1.073)	1.305 (0.956–1.780)	0.093
Female sex	0.949 (0.469–1.923)	–	0.885
BMI	1.068 (1.014–1.126)	1.549 (1.094–2.193)	0.014
Drinking	2.099 (0.809–5.444)	–	0.127
Smoking	1.680 (0.682–4.138)	–	0.259
Family pressure	1.606 (1.034–2.495)	1.516 (1.030–2.232)	0.035
Peer pressure	1.012 (0.623–1.642)	1.011 (0.646–1.582)	0.962
Media pressure	1.324 (0.903–1.942)	1.351 (0.897–2.035)	0.150
Thin-ideal internalization	0.874 (0.537–1.425)	0.882 (0.558–1.393)	0.590
Muscular internalization	1.275 (0.852–1.909)	1.266 (0.855–1.874)	0.238
GAD-7	1.021 (0.934–1.116)	1.116 (0.700–1.780)	0.644
PHQ-9	1.064 (0.979–1.157)	1.421 (0.886–2.278)	0.145
BAS-2	0.682 (0.460–1.013)	0.719 (0.511–1.011)	0.058

Model 3 simultaneously includes all predictors shown and is the primary inferential model. OR, odds ratio; CI, confidence interval; SD, sample standard deviation.

### Food addiction symptom count: full negative-binomial model

In the full negative-binomial model, family pressure and PHQ-9 were positively associated with symptom count, whereas BAS-2 was inversely associated (Table 3). Per SD, their IRRs were 1.171 (95% CI 1.023–1.340; *p* = 0.022), 1.284 (95% CI 1.087–1.518; *p* = 0.003), and 0.883 (95% CI 0.785–0.992; *p* = 0.037). BMI and GAD-7 were not statistically significant, and no other predictor reached statistical significance in the full model (Figure 1).

**Table 3:** Full negative-binomial model for food addiction symptom count.

Predictor	IRR/unit (95% CI)	IRR/SD (95% CI)	*P*
Age	1.003 (0.990–1.017)	1.026 (0.919–1.146)	0.650
Female sex	0.814 (0.634–1.044)	–	0.105
BMI	1.007 (0.990–1.023)	1.045 (0.936–1.167)	0.430
Drinking	1.218 (0.874–1.696)	–	0.244
Smoking	0.905 (0.653–1.252)	–	0.545
Family pressure	1.197 (1.026–1.396)	1.171 (1.023–1.340)	0.022
Peer pressure	1.083 (0.913–1.284)	1.076 (0.919–1.260)	0.360
Media pressure	1.002 (0.875–1.147)	1.002 (0.867–1.158)	0.976
Thin-ideal internalization	0.994 (0.838–1.179)	0.994 (0.847–1.167)	0.945
Muscular internalization	0.985 (0.855–1.134)	0.985 (0.859–1.130)	0.829
GAD-7	0.999 (0.968–1.031)	0.997 (0.845–1.175)	0.968
PHQ-9	1.045 (1.015–1.077)	1.284 (1.087–1.518)	0.003
BAS-2	0.865 (0.756–0.991)	0.883 (0.785–0.992)	0.037

Model 3 simultaneously includes all predictors shown and is the primary inferential model. Negative-binomial regression was used because the Poisson model was overdispersed. IRR, incidence rate ratio; CI, confidence interval; SD, sample standard deviation.

**Figure 1:**
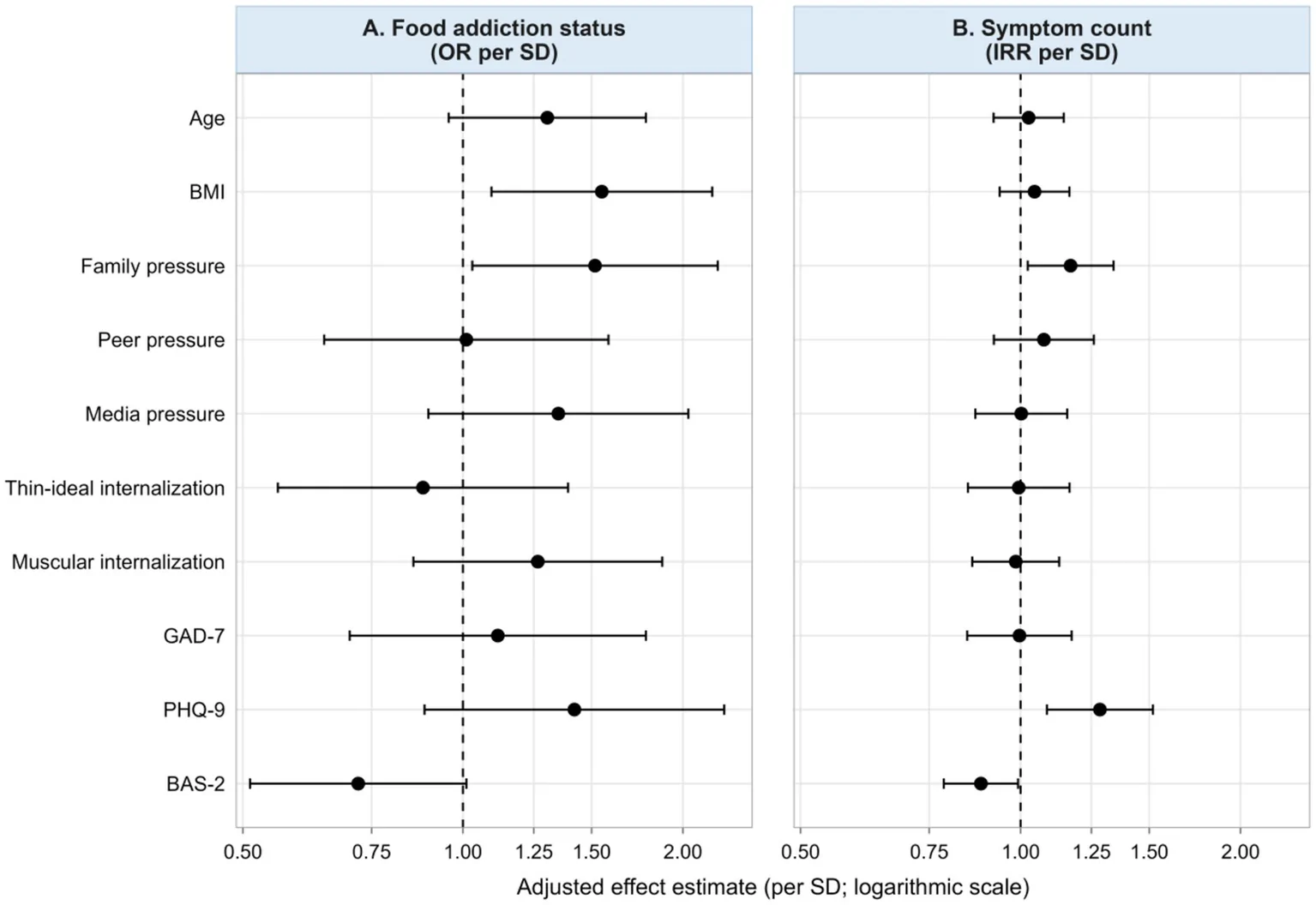
Standardized associations in the full models. Points represent adjusted ORs (FA status) or IRRs (symptom count) per sample SD in Model 3; lines represent 95% CIs.

### Exploratory hierarchical comparisons and model evaluation

Exploratory hierarchical comparisons showed that Model 2 fit both FA status and symptom count better than Model 1 (both likelihood-ratio tests *p* < 0.001). Adding peer pressure, media pressure, appearance-ideal internalization and GAD-7 in Model 3 did not significantly improve fit over Model 2 for FA status (*p* = 0.350) or symptom count (*p* = 0.946). Complete Model 1 and Model 2 results are reported in Supplementary Tables S1, S2, and model-fit indices in Supplementary Table S5.

Family pressure was the only pressure source with a statistically significant independent coefficient in the primary models; however, the family pressure, peer pressure, and media pressure coefficients were not statistically distinguishable (FA status: Wald χ^2^ = 1.435, df = 2, *p* = 0.488; symptom count: Wald χ^2^ = 2.695, df = 2, *p* = 0.260). PHQ-9 and GAD-7 were strongly correlated (Spearman *r* = 0.721), and all VIFs were below 3. Therefore, the results support neither a hierarchy among pressure sources nor a clear dissociation between depression and anxiety (Supplementary Tables S3, S4).

The primary full logistic model had an apparent AUC of 0.761 and a Brier score of 0.191. After internal validation with 1,000 bootstrap resamples, the optimism-corrected AUC was 0.707 (bootstrap interval 0.650–0.768), the corrected Brier score was 0.216 (0.190–0.248), the corrected calibration slope was 0.734 (0.530–0.989), and the corrected calibration intercept was 0.013 (−0.317 to 0.352). The model retained moderate discrimination after internal correction, although external validation remains necessary before clinical prediction or screening use (Supplementary Table S5).

## Discussion

In this sample of 240 Chinese candidates for bariatric surgery, the prevalence of food addiction was 39.6%, within the broad range of 6.3 to 69% reported in previous studies. Consistent with our hypothesis, patients with FA had higher BMI, greater psychological distress, more pronounced sociocultural appearance pressure from all sources, and lower body appreciation. In the full logistic model, BMI and family pressure were associated with FA status, whereas PHQ-9 and BAS-2 were not statistically significant. In the full negative-binomial model, family pressure and PHQ-9 were positively associated with symptom count and BAS-2 was inversely associated.

### Sources of appearance pressure

Family, peer, and media pressure are conceptually interrelated components of the Tripartite Influence Model, which identifies the three as major sociocultural sources of body dissatisfaction and disordered eating (van den Berg et al., 2002; Paterna et al., 2021). However, increasing evidence suggests that the relative contribution of each source may vary across populations and life stages. In a study of Italian adolescents, family pressure was directly associated with body dissatisfaction and eating-disorder symptoms, whereas peer pressure operated indirectly through appearance comparison (Iannattone et al., 2025). Similarly, among pregnant women, only media and family pressure were directly associated with body dissatisfaction, whereas peer pressure showed no significant direct or indirect effect (Di Gesto et al., 2025). In the full models, family pressure was the only pressure source with a statistically significant independent coefficient. However, formal omnibus and pairwise comparisons showed that its coefficient did not differ significantly from those of peer or media pressure. Thus, the findings support family pressure as a correlate in this sample but do not establish its statistical primacy. Cultural interpretations concerning family interdependence remain hypotheses requiring longitudinal or mechanistic testing (Lawson et al., 2020).

### Depression, anxiety and body appreciation

The PHQ-9 association in the full models was outcome-specific: it was statistically significant for symptom count but not for binary FA status. Depressive symptoms are not only associated with FA but also predict poorer postoperative outcomes (Ramirez et al., 2023; Jacobs et al., 2024; Dawes et al., 2016). GAD-7 was not statistically significant in either primary model. However, PHQ-9 and GAD-7 were strongly correlated and their standardized coefficients were not statistically distinguishable; the findings should therefore not be interpreted as showing that depression is more important than anxiety. Depression-related mechanisms discussed in previous studies, including negative reinforcement and difficulties with self-regulation, remain plausible, but they were not measured as mediators in this study. Higher BAS-2 scores were associated with fewer FA symptoms, but the estimate for the binary outcome was not statistically significant. This difference may reflect information loss caused by dichotomizing the symptom count (Ivezaj and Grilo, 2018).

### Clinical interpretation

The measures used in this study may help characterize the psychosocial complexity of candidates for bariatric surgery. After bootstrap correction, the primary full logistic model retained moderate discrimination, supporting its descriptive value while also indicating that its performance should be confirmed in independent samples. Given the relevance of psychological and behavioral factors to bariatric surgery outcomes, comprehensive preoperative assessment may reasonably consider eating symptoms, mood, body image and interpersonal appearance pressure (Sogg et al., 2016). Given prospective evidence of postoperative alcohol-related risk, longitudinal monitoring of alcohol and other substance use may also be relevant; however, our preoperative binary drinking variable cannot support prediction of such outcomes. Future prospective studies may further determine whether these correlates improve prediction or guide targeted psychological support in clinical practice.

### Food addiction

FA remains a debated construct rather than an independent clinical diagnosis. One perspective conceptualizes addictive-like eating as a substance-based response to specific foods, whereas an alternative eating-addiction perspective locates the addictive process in eating itself rather than in particular food constituents (Hebebrand et al., 2014; Schulte et al., 2017). YFAS-defined FA also overlaps substantially with binge-eating disorder, although this overlap does not indicate that the two constructs are entirely identical (Burrows et al., 2017). Accordingly, our findings should be interpreted as associations with a YFAS 2.0-defined phenotype, rather than as evidence demonstrating the existence of an addictive disorder or a specific neurobiological mechanism.

For bariatric surgery populations, another addiction-related concern is the possible emergence or worsening of alcohol-, tobacco-, or other substance-related problems after surgery. Prospective studies have reported increases in symptoms of alcohol use disorder, particularly following Roux-en-Y gastric bypass (King et al., 2012; King et al., 2017), whereas direct evidence that remission of preoperative FA leads to addiction transfer remains inconsistent (Dickhut et al., 2021). In the present study, drinking and smoking were included only as binary preoperative covariates; the cross-sectional data cannot evaluate postoperative addiction transfer or cross-addiction. Longitudinal follow-up using measures of alcohol and substance use is needed.

## Limitations

First, the cross-sectional design precludes temporal or causal inference; reverse or bidirectional associations are possible. Second, this was a single-center sample of Chinese candidates for bariatric surgery, limiting generalizability to other clinical and regional populations. Third, questionnaire data were self-reported and may be subject to bias. Excluding questionnaires that failed the attention check or were completed too quickly helped control data quality but may also have introduced selection bias. Fourth, binge-eating symptoms and weight stigma were not measured. These constructs may help explain the associations among appearance pressure, psychological distress and FA (di Giacomo et al., 2022; Levinson et al., 2024). Fifth, drinking and smoking were recorded only as binary preoperative variables, and postoperative substance-use outcomes were unavailable; this study therefore cannot test the addiction-transfer or cross-addiction hypotheses.

## Conclusion

Among Chinese candidates for bariatric surgery, the full models showed that BMI and family pressure were associated with binary FA status, whereas family pressure, depressive symptoms, and body appreciation were associated with symptom count. Although family pressure was the only pressure source with a statistically significant independent coefficient, formal tests showed no differences among the family, peer, and media pressure coefficients. The PHQ-9 and BAS-2 findings also differed by outcome. These findings suggest that food addiction is associated with several important psychosocial characteristics among Chinese candidates for bariatric surgery, but prospective studies are needed to clarify these associations and their potential mechanisms.

## Data Availability

The raw data supporting the conclusions of this article will be made available by the authors, without undue reservation.

## Footnotes

1
https://www.wjx.cn/
