# Supplementary material for: Multidimensional psychosocial correlates of food addiction status and symptom count in Chinese candidates for bariatric surgery: a cross-sectional study
Source: Front Psychol. 2026 Sep 30;17:1942332. doi: 10.3389/fpsyg.2026.1942332 (PMC13662120; doi:10.3389/fpsyg.2026.1942332)
Supplement: Supplementary file 1 [file Table_1.docx]

**Supplementary Files for**

**Multidimensional Psychosocial Correlates of Food Addiction Status and Symptom Count in Chinese Candidates for Bariatric Surgery: A Cross-Sectional Study**

**Supplementary Table S1.** Exploratory hierarchical logistic models for food addiction status

| **Predictor** | **Model 1 OR/unit (95% CI)** | **Model 1 OR/SD (95% CI)** | **P** | **Model 2 OR/unit (95% CI)** | **Model 2 OR/SD (95% CI)** | **P** |
| --- | --- | --- | --- | --- | --- | --- |
| Age | 1.033 (0.998-1.069) | 1.304 (0.983-1.730) | 0.065 | 1.036 (0.998-1.075) | 1.333 (0.984-1.805) | 0.063 |
| Female sex | 0.907 (0.479-1.717) | - | 0.765 | 0.875 (0.440-1.740) | - | 0.704 |
| BMI | 1.065 (1.017-1.114) | 1.517 (1.122-2.050) | 0.007 | 1.062 (1.010-1.117) | 1.490 (1.066-2.082) | 0.019 |
| Drinking | 2.649 (1.132-6.202) | - | 0.025 | 2.376 (0.940-6.003) | - | 0.067 |
| Smoking | 1.697 (0.749-3.846) | - | 0.205 | 1.576 (0.659-3.767) | - | 0.307 |
| Family pressure | - | - | - | 1.898 (1.335-2.697) | 1.755 (1.289-2.391) | <0.001 |
| PHQ-9 | - | - | - | 1.080 (1.021-1.142) | 1.543 (1.124-2.118) | 0.007 |
| BAS-2 | - | - | - | 0.709 (0.488-1.029) | 0.743 (0.539-1.025) | 0.070 |

Note. Model 1 includes age, sex, BMI, drinking and smoking. Model 2 additionally includes family pressure, PHQ-9 and BAS-2. OR, odds ratio; CI, confidence interval; SD, sample standard deviation.

**Supplementary Table S2.** Exploratory hierarchical negative-binomial models for food addiction symptom count

| **Predictor** | **Model 1 IRR/unit (95% CI)** | **Model 1 IRR/SD (95% CI)** | **P** | **Model 2 IRR/unit (95% CI)** | **Model 2 IRR/SD (95% CI)** | **P** |
| --- | --- | --- | --- | --- | --- | --- |
| Age | 1.006 (0.991-1.020) | 1.046 (0.931-1.176) | 0.449 | 1.004 (0.990-1.017) | 1.029 (0.923-1.148) | 0.605 |
| Female sex | 0.835 (0.639-1.092) | - | 0.189 | 0.817 (0.639-1.046) | - | 0.109 |
| BMI | 1.012 (0.994-1.030) | 1.084 (0.963-1.219) | 0.180 | 1.006 (0.990-1.023) | 1.042 (0.934-1.161) | 0.462 |
| Drinking | 1.417 (0.997-2.015) | - | 0.052 | 1.241 (0.898-1.717) | - | 0.191 |
| Smoking | 0.922 (0.649-1.308) | - | 0.648 | 0.913 (0.664-1.255) | - | 0.575 |
| Family pressure | - | - | - | 1.245 (1.102-1.408) | 1.213 (1.089-1.351) | <0.001 |
| PHQ-9 | - | - | - | 1.044 (1.024-1.065) | 1.279 (1.146-1.427) | <0.001 |
| BAS-2 | - | - | - | 0.865 (0.758-0.987) | 0.882 (0.787-0.989) | 0.031 |

Note. Model 1 includes age, sex, BMI, drinking and smoking. Model 2 additionally includes family pressure, PHQ-9 and BAS-2. IRR, incidence rate ratio; CI, confidence interval; SD, sample standard deviation.

**Supplementary Table S3.** Formal coefficient comparisons

| **Test** | **Outcome** | **Hypothesis/Contrast** | **Statistic or ratio (95% CI)** | **df** | **P** | **FDR P** |
| --- | --- | --- | --- | --- | --- | --- |
| Omnibus equality | FA status | family pressure = peer pressure = media pressure | Wald χ² = 1.435 | 2 | 0.488 | - |
| Omnibus equality | Symptom count | family pressure = peer pressure = media pressure | Wald χ² = 2.695 | 2 | 0.260 | - |
| Pairwise | FA status | family pressure vs peer pressure | 1.588 (0.743-3.393) | - | 0.233 | 0.525 |
| Pairwise | FA status | family pressure vs media pressure | 1.213 (0.669-2.199) | - | 0.525 | 0.525 |
| Pairwise | FA status | peer pressure vs media pressure | 0.764 (0.370-1.576) | - | 0.466 | 0.525 |
| Pairwise | Symptom count | family pressure vs peer pressure | 1.105 (0.849-1.439) | - | 0.458 | 0.551 |
| Pairwise | Symptom count | family pressure vs media pressure | 1.194 (0.966-1.476) | - | 0.101 | 0.302 |
| Pairwise | Symptom count | peer pressure vs media pressure | 1.081 (0.837-1.394) | - | 0.551 | 0.551 |
| Exploratory comparison | FA status | PHQ-9 vs GAD-7 (per SD) | 1.273 (0.531-3.052) | - | 0.589 | - |
| Exploratory comparison | Symptom count | PHQ-9 vs GAD-7 (per SD) | 1.289 (0.945-1.757) | - | 0.109 | - |

Note. Pressure-source comparisons are on the original one-point SATAQ-4 subscale scale and directly test equality of the Model 3 family, peer, and media pressure coefficients. PHQ-9 versus GAD-7 comparisons use standardized coefficients and are exploratory because the two measures are strongly correlated. A ratio of effect ratios equal to 1 indicates equal coefficients.

**Supplementary Table S4.** Predictor correlation matrix and variance inflation factors

| **Predictor** | **Age** | **BMI** | **Family pressure** | **Peer pressure** | **Media pressure** | **Thin-ideal internalization** | **Muscular internalization** | **GAD-7** | **PHQ-9** | **BAS-2** | **VIF logistic** | **VIF NB** |
| --- | --- | --- | --- | --- | --- | --- | --- | --- | --- | --- | --- | --- |
| Age | 1.00 | -0.21 | -0.06 | 0.02 | -0.02 | -0.07 | 0.02 | 0.01 | 0.09 | 0.02 | 1.15 | 1.12 |
| BMI | -0.21 | 1.00 | 0.09 | -0.00 | -0.04 | -0.06 | -0.03 | 0.05 | -0.05 | -0.03 | 1.21 | 1.14 |
| Family pressure | -0.06 | 0.09 | 1.00 | 0.60 | 0.45 | 0.50 | 0.31 | -0.04 | -0.01 | 0.04 | 1.63 | 1.68 |
| Peer pressure | 0.02 | -0.00 | 0.60 | 1.00 | 0.59 | 0.58 | 0.40 | -0.05 | -0.02 | 0.03 | 2.30 | 2.30 |
| Media pressure | -0.02 | -0.04 | 0.45 | 0.59 | 1.00 | 0.54 | 0.40 | 0.06 | 0.07 | -0.04 | 1.85 | 1.93 |
| Thin-ideal internalization | -0.07 | -0.06 | 0.50 | 0.58 | 0.54 | 1.00 | 0.53 | 0.01 | 0.00 | 0.01 | 2.42 | 2.35 |
| Muscular internalization | 0.02 | -0.03 | 0.31 | 0.40 | 0.40 | 0.53 | 1.00 | -0.04 | -0.09 | 0.19 | 1.85 | 1.77 |
| GAD-7 | 0.01 | 0.05 | -0.04 | -0.05 | 0.06 | 0.01 | -0.04 | 1.00 | 0.72 | -0.41 | 2.48 | 2.78 |
| PHQ-9 | 0.09 | -0.05 | -0.01 | -0.02 | 0.07 | 0.00 | -0.09 | 0.72 | 1.00 | -0.39 | 2.55 | 2.80 |
| BAS-2 | 0.02 | -0.03 | 0.04 | 0.03 | -0.04 | 0.01 | 0.19 | -0.41 | -0.39 | 1.00 | 1.30 | 1.28 |
| Female sex | - | - | - | - | - | - | - | - | - | - | 1.31 | 1.31 |
| Drinking | - | - | - | - | - | - | - | - | - | - | 1.07 | 1.12 |
| Smoking | - | - | - | - | - | - | - | - | - | - | 1.20 | 1.22 |

Note. Matrix entries are Spearman correlations among continuous Model 3 predictors; dashes are shown for categorical predictors. VIFs are reported for every predictor from the fully adjusted logistic and negative-binomial (NB) models.

**Supplementary Table S5.** Exploratory model fit, overdispersion check, and bootstrap internal validation

| **Panel** | **Outcome** | **Model** | **Estimate 1** | **Estimate 2** | **Additional metric** |
| --- | --- | --- | --- | --- | --- |
| A: Model fit | FA status | Model 1 | AIC 316.19 | BIC 337.07 | AUC 0.666; Brier 0.222 |
| A: Model fit | FA status | Model 2 | AIC 295.20 | BIC 326.53 | AUC 0.747; Brier 0.197; LRT vs Model 1 P <0.001 |
| A: Model fit | FA status | Model 3 | AIC 299.63 | BIC 348.36 | AUC 0.761; Brier 0.191; LRT vs Model 2 P 0.350 |
| A: Model fit | Symptom count | Model 1 | AIC 1226.76 | BIC 1251.12 | Theta 1.788 |
| A: Model fit | Symptom count | Model 2 | AIC 1194.46 | BIC 1229.26 | Theta 2.421; LRT vs Model 1 P <0.001 |
| A: Model fit | Symptom count | Model 3 | AIC 1203.27 | BIC 1255.48 | Theta 2.439; LRT vs Model 2 P 0.946 |
| B: Diagnostic | As specified | Poisson dispersion statistic | 2.495 | - | - |
| C: Bootstrap | FA status | Optimism-corrected AUC | 0.707 | 0.650-0.768 | 1,000 resamples |
| C: Bootstrap | FA status | Optimism-corrected Brier | 0.216 | 0.190-0.248 | 1,000 resamples |
| C: Bootstrap | FA status | Corrected calibration slope | 0.734 | 0.530-0.989 | 1,000 resamples |
| C: Bootstrap | FA status | Corrected calibration intercept | 0.013 | -0.317-0.352 | 1,000 resamples |

Note. Model 3 is the primary full model; Models 1 and 2 are exploratory nested comparisons. The Poisson dispersion statistic supports negative-binomial regression for symptom count. Bootstrap intervals are empirical 2.5th–97.5th percentiles across 1,000 resamples of the primary full logistic model.
